# Developing an automatic decision‐assistance tool to choose proton/photon radiotherapy for patients with prostate cancer

**DOI:** 10.1002/acm2.70497

**Published:** 2026-02-18

**Authors:** Mengyang Li, Linyi Shen, Xinyuan Chen, Guiyuan Li, Jialin Ding, Kuo Men, Junlin Yi, Jianrong Dai

**Affiliations:** ^1^ National Cancer Center/National Clinical Research Center for Cancer/Cancer Hospital Chinese Academy of Medical Sciences and Peking Union Medical College Beijing China

**Keywords:** automatic decision method, deep learning, NTCP, photon therapy, prostate cancer, proton therapy

## Abstract

**Purpose:**

It is important to guide staff in choosing appropriately between photon and proton radiotherapy. This study develops an automatic decision method to select the most clinically beneficial radiotherapy technique (proton or photon) for patients with prostate cancer. An automatic decision method was developed to help staff in choosing appropriately between photon and proton radiotherapy for patients with prostate cancer.

**Materials and Methods:**

Forty‐eight patients with prostate cancer were enrolled. First, photon and proton dose prediction models (*M*_ph_ and *M*_pr_) were trained using reference plans from previous patients’ data. Second, the predicted values of *V*_6300cGy_ (rectum wall) were obtained using the trained models, *M*_ph_ and *M*_pr_, and these values were used to calculate the Normal Tissue Complication Probability (NTCP). Finally, if the photon NTCP exceeded 10%, the proton NTCP was calculated, and the difference (ΔNTCP) between the two was used to guide treatment selection. The accuracy of the decision support system was evaluated by comparing dose distributions, NTCPs, and decision outcomes between manual and automatic plans using paired *t*‐tests.

**Results:**

The deep learning (DL) model showed a mean absolute error (MAE) of 4.60 ± 1.80 for the rectum wall in the photon group and 3.64 ± 1.27 in the proton group. There was no statistically significant difference in *V*_6300cGy_ (rectum wall) between manual plans and model predictions (photon group *p* = 0.594, proton group *p* = 0.057). Similarly, no significant differences were observed in NTCP values for the rectum wall (photon group *p* = 0.383, proton group *p* = 0.100). The system correctly predicted the treatment modality in 45 of 48 cases, resulting in an accuracy rate of 93.75%, with AUC values for the decision method at 0.88.

**Conclusion:**

The proposed automatic decision method matches dose distributions, accurately calculates NTCPs, and supports precise radiotherapy technique selection, enhancing the clinical efficiency.

**Abbreviations**
AUC: Area under the curve
CNN: Convolutional Neural Networks
CTV: Clinical target volume
DBA: Deeper bottleneck architectures
DDM: Dose Distribution Map
DL: Deep learning
GT: Ground truth
IMRT: Intensity‐modulated radiation therapy
MAE: Mean absolute error
NTCP: Normal Tissue Complication Probability
OAR: Organs at risk
PT: Proton therapy
PTV: Planning target volume
ROC: Receiver operating characteristic
ROI: Regions of interest
TPS: Treatment planning system
VMAT: Volumetric modulated arc therapy
AI: Artificial Intelligence
IMPT: Intensity‐modulated proton therapy

## 1 Introduction

Prostate cancer is the second most commonly diagnosed cancer among male patients.^1^ It can lead to death and significantly impact the quality of life.^2^, ^3^ Radiotherapy (RT) accounts for approximately 30% or more of prostate cancer treatments and is the second most common treatment modality after surgery.^4^

Photon and proton therapies are two notable modalities for radiotherapy.^5^ In photon therapy, volumetric modulated arc therapy (VMAT) and intensity‐modulated radiation therapy (IMRT) are the preferred techniques for treating prostate cancer and are widely used in clinical practice.^6^ Numerous studies have shown that both techniques deliver a high dose to the target area while maximizing the sparing of adjacent organs at risk (OARs).^7^, ^9^

Due to the Bragg peak characteristic, proton therapy (PT) for prostate cancer achieves sharp dose gradients at the target's edge, theoretically protecting normal tissues. PT can reduce the integral dose compared to conventional photon‐based RT while maintaining a highly conformal dose to the tumor target for some patients.^10^ However, PT has several drawbacks compared to photon therapy: (1) Patients face a greater direct financial burden with PT.^11^, ^12^, ^13^ (2) The number of PT equipment is much lower than that of photon therapy equipment. (3) Not all prostate cancer patients derive significantly greater clinical benefits from PT than from photon therapy. Therefore, it is crucial to determine whether a prostate cancer patient needs PT. Existing treatment plan comparison methods include dose‐volume parameter analysis, such as dose‐volume histograms (DVH), isodose curve, among others. Additionally, the normal tissue complication probability (NTCP) method is used in research.

NTCP predicts the risk of treatment‐related complications based on radiation doses and volume information about OARs.^14^ Recently, studies have reported using NTCP models to estimate the risk of RT‐related morbidity and to compare photon‐based RT with PT.^15^, ^16^ Traditionally, NTCP values are calculated after manually designing RT plans based on photon or proton techniques, a process that is time‐consuming.

Deep learning (DL) can generate dose distribution maps(DDMs) for both photon and proton plans in a few seconds.^17^, ^18^, ^19^ By learning from large datasets of medical images and dose data, Convolutional neural networks (CNN) can significantly improve the efficiency of RT planning and reduce clinical workload.^20^ For example, ResNet can accurately and efficiently predict radiation doses to guide automated planning.^18^, ^19^

An automatic decision‐assistance tool for selecting patients for PT using NTCP with DL‐based dose prediction has been proposed in the past 2 years.^15^, ^21^ However, the methodology and clinical application of automatic decision‐making for prostate cancer still require in‐depth and extensive research.

This study aimed to establish a decision‐assistance tool to determine which technique (photon or proton) is suitable for prostate cancer patients. Previous research^14^, ^18^, ^20^ on RT planning for prostate cancer has mostly confined the clinical target volume (CTV) to the prostate itself or seminal vesicles, with NTCP values often derived from manual plans. In contrast, this study focused on patients whose radiation fields encompass lymphatic drainage regions and introduced dose prediction technology based on a DL model.

The contribution of this study lies in its coverage of a broader range of prostate cancer target volumes, enabling adaptability to a wider array of clinical scenarios. The use of DL in decision‐making enhances the treatment efficiency of clinical institutions, expediting access to clinical services for a larger patient population. Additionally, the proton/photon decision‐assistance tool facilitates the selection of more suitable clinical treatment techniques, ultimately helping reduce radiation therapy‐related complications.

## 2 Material and methods

### 2.1 Patient cohort

A total of 48 prostate cancer patients who received VMAT with full arc at our institution from 2018 to 2023 were retrospectively collected for this study. The mean age of the patients was 66.55 ± 7.56 years old. The use of patient data was approved by the Institutional Ethical Review Board of the National Cancer Center. Intensity‐modulated proton therapy (IMPT) plans were specifically designed for each patient. Pencil beam scanning technology was employed in the IMPT, the range shifter was RS4cm, and the spot spacing was 0.6 cm. The prescribed dose for these prostate cancer patients was 67.5 Gy [RBE] in 25 fractions. All patients were categorized into three groups based on their treatment plans.

The CTV included the prostate, seminal vesicles, and the posterior wall of the bladder. For dual‐dose gradient treatment plans, the CTV also encompassed the pelvic lymphatic drainage area. The planning target volume (PTV) was defined as follows: a 1.0 cm margin was applied in the superior, inferior, left, right, and anterior directions and a 0.5 cm margin in the posterior direction. For treatment plans including lymphatic drainage areas, a 0.7 cm margin was applied in the anterior, left, and right directions, a 0.5 cm margin in the posterior direction, and a 1.0 cm margin in the superior and inferior directions (Table 1).

**TABLE 1:** The expansion margins in all directions for PTV1 and PTV2. PTV1 is generated by expanding the CTV encompassing the prostate and seminal vesicles, while PTV2 is generated by expanding the CTV encompassing the pelvic lymph nodes.

Margin	PTV1	PTV2
Superior	1.0 cm	1.0 cm
Inferior	1.0 cm	1.0 cm
Left	1.0 cm	0.7 cm
Right	1.0 cm	0.7 cm
Anterior	1.0 cm	0.7 cm
Posterior	0.5 cm	0.5 cm

A single‐dose gradient of 67.5 Gy was delivered to the prostate and seminal vesicles, excluding the lymph nodes. This target volume is designated as PTV1. For plans that included the pelvic lymph nodes, a double‐dose gradient was used, delivering 67.5 Gy to the prostate and seminal vesicles combined with either 50.0 or 45.0 Gy to the pelvic lymph nodes. The pelvic lymph nodes are designated as PTV2 in the planning system. The dose prescription and compliance criteria are listed in Table 2.

**TABLE 2:** Overview of the clinical dose criteria. The goal for PTV1 and PTV2 was to deliver 6750cGy and 4500cGy (or 5000cGy) to at least 95% of their respective volumes, denoted as V6750cGy ≥ 95% for PTV1 and V4500cGy ≥ 95% (or V5000cGy ≥ 95%) for PTV2. When considered infeasible, the 95% coverage criterion was adjusted to 90%. Regarding OARs, the key requirement was to limit the maximum dose received and the relative volume of the organ exposed to reference doses within specified constraints.

ROI	Dosimetric parameter	Per protocol	Variation acceptable
PTV1	*V*_6750cGy_ [%]	≥95	≥90–< 95
PTV2	^*^ *V*_4500–5000cGy_ [%]	≥95	≥90–< 95
Rectum wall	*D*_max_ [cGy]	<7000	<7000
Rectum	*D*_max_ [cGy]	<7000	<7000
	*V*_5000cGy_ [%]	<30	<30
	*V*_6000cGy_ [%]	<15	<15
Bladder	*D*_max_ [cGy]	<7100	<7100
	*V*_5000cGy_ [%]	<30	<30
	*V*_6000cGy_ [%]	<20	<20
Femur L	*V*_5000cGy_ [%]	<50	<50
Femur R	*V*_5000cGy_ [%]	<50	<50
Colon	*D*_max_ [cGy]	<5400	<5400
	*V*_5000cGy_ [%]	<10	<10
Intestine	*D*_max_ [cGy]	<5200	<5200
	*V*_5000cGy_ [%]	<5	<5
	*V*_4500cGy_ [%]	<5	<5

*Note*: ^*^ *V*_4500–5000cGy_ [%] means the percentage volume that receives a radiation dose of either 4500 cGy or 5000 cGy.

Individual treatment plans were designed for each patient based on their unique anatomies, with beam angles set at 90°, 270°, 150°, and 210°.

### 2.2 General workflow

**FIGURE 1:**
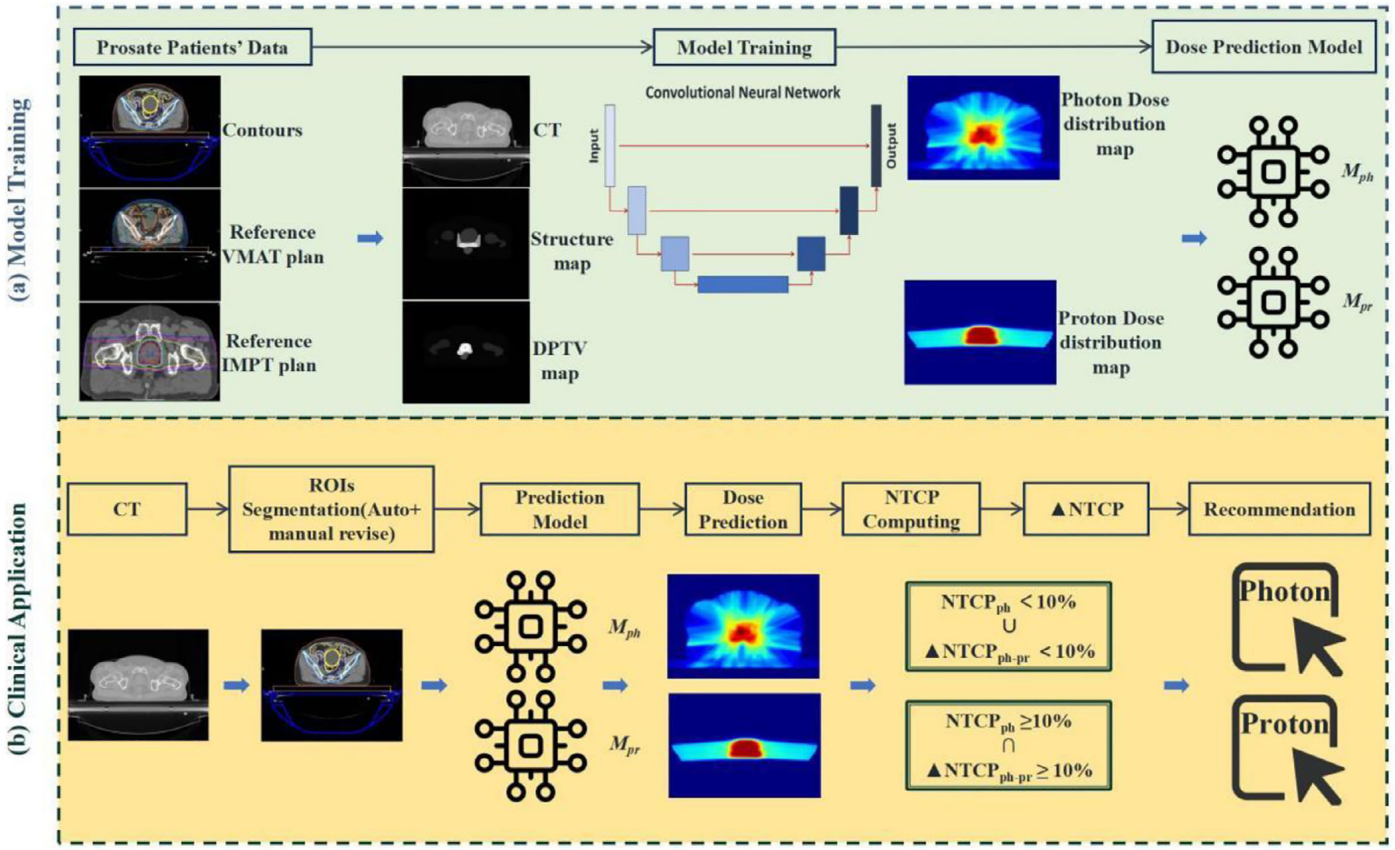
The overall workflow for the decision‐making methodology in proton/photon RT encompasses two primary stages. Initially, we leverage prior patient data, comprising simulated CT contours and reference plans, to train predictive models for proton (*M*_pr_) and photon (*M*_ph_). Upon direct input of a patient's CT image, automated segmentation of ROIs was performed, followed by manual refinement for accuracy. Utilizing the trained models *M*_ph_ and *M*_pr_, dose distributions were predicted, and the resultant dose parameters were subjected to NTCP calculations. When the photon NTCP fell below 10%, photon therapy was preliminarily selected. Subsequently, the difference in NTCP between photon and proton (ΔNTCP) was evaluated; if ΔNTCP was ≥ 10%, PT was favored; otherwise, photon therapy remains the choice.

The proposed decision‐making workflow for selecting proton or photon RT for patients, as outlined in this article, consists of two primary steps (Figure 1). First, a proton dose prediction model (*M*_pr_) and a photon dose prediction model (*M*_ph_) are trained using historical patient data from proton and photon therapies. The training data includes simulated CT contours, along with IMPT, and VMAT reference plans.

DL technology was used to automatically segment the CT images and generate the planned tumor volume and organs at risk. The dose distribution was then predicted using a CNN. Both CT segmentation and dose distribution prediction DL tools were developed by our institution. The predicted dose was applied to the NTCP model to calculate the predicted NTCP value. A two‐step threshold approach was adopted to choose between proton and photon therapy.

The decision‐making process involved two steps:

Step 1: The NTCP value for photon therapy was evaluated. If it exceeded 10%, the process proceeded to step 2; otherwise, photon therapy was directly selected.
Step 2: A comparison between proton and photon technologies was conducted. Photon therapy was considered if ΔNTCP ≤ 10%;^13^ otherwise, PT was chosen.

Dose and volume information, as well as the treatment planning system (TPS) NTCP value, were calculated using the Pinnacle 16.2 TPS for each clinical case. Verification that the DL‐based method yielded NTCP values comparable to TPS was achieved by comparing the predicted and calculated ΔNTCP. The feasibility of this ΔNTCP‐assisted approach for technique selection was supported by previous studies.^15^, ^22^, ^23^

### 2.3 DL model for dose prediction

**FIGURE 2:**
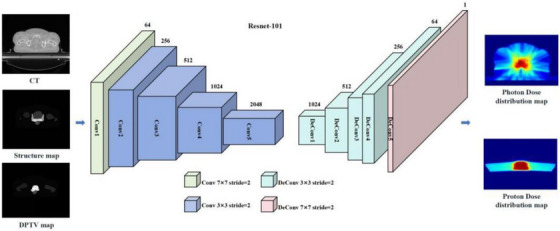
DL network for dose prediction. The training workflow for the COM model encompasses 101 iterations, utilizing three crucial input channels: CT images, structure maps, and DPTV maps. The output generated is the DDM for both photon and proton therapies, which undergoes interpolation to ensure that the DDM's pixel resolution aligns perfectly with that of input images. The underlying DL architecture builds upon ResNet‐101, employing an encoder‐decoder configuration. The encoder, comprising light green and blue segments featuring a bottleneck, works in concert with the decoder, and constituted by cyan and pink parts.

A DL network for dose prediction task is shown in Figure 2. This study employed a combined anatomical mapping approach, utilizing DPTV maps, structure maps, and CT scans as three‐channel inputs for training a deep‐learning‐based Combined (COM) model. This model aimed to predict corresponding DDMs. At the core of the network architecture lies a Resnet with 101 layers, which was explored in our research.^16^ The initial layer, Conv1, includes a 7 × 7 convolutional layer with 64 filters, followed by a max‐pooling operation for dimensionality reduction. Conv2, Conv3, Conv4, and Conv5 consist of 3, 4, 23, and 5 deeper bottleneck architectures (DBAs), respectively.^23^ Each DBA includes three convolutional layers with 1 × 1, 3 × 3, and 1 × 1 configurations, along with residual connections. The output of Conv5 is a downscaled version of the original image, with the resolution reduced to 1/8th. To restore the original image resolution, an upsampling technique based on fractionally‐strided deconvolution was employed. Model training and testing were conducted using PyTorch on a workstation with an Intel® Core i7 CPU (3.4 GHz) and a TITAN XP graphics card.

To evaluate the performance of the DL method for dose prediction, a fivefold cross‐validation approach was used. Forty‐eight patient cases were randomly divided into five equal‐sized subsets. In each validation, the model was trained on four subsets (80% of the data) and tested on the remaining subset (20% of the data). This process was repeated five times, with a different subset used for testing each time and the remaining subsets used for training. This resulted in five sets of results that comprehensively assessed the performance of the DL method. DDMs of approved plans were used as ground truth (GT) for model estimation.

### 2.4 Probability of complications

The Lyman–Kutcher–Burman model was taken from the original publication.^24^

(1) $$\text{NTCP}\;=\frac{1}{\sqrt{2\pi }}\underset{-\infty }{\int^{u}}\;e^{\frac{-t^{2}}{2}}dt$$

A logistics model based on DVH was adopted to calculate NTCP value.

(2) $$\text{NTCP}\;=\frac{1}{1-e^{-S}}$$

where S is calculated according to the equation (3) proposed by Pedersen J et al.^14^

(3) $$\begin{array}{cc} S & =-3.07+0.79*\left(\text{anticoagulant use}\right)\\ & \;+0.12*\left(\text{Rectum Wall}\left(V_{6300\mathrm{cGy}}\right)\right) \end{array}$$

The prediction parameter of Rectum Wall dose volume percentage was transformed to *V*_6300cGy_ by the biological effective dose transform formula.

### 2.5 Decision‐making criteria

First, the NTCP value associated with photon therapy was assessed. If this value exceeds 10%, a comparative analysis between proton and photon technologies was conducted. If ΔNTCP ≤ 10%, photon therapy was preferred; otherwise, PT was selected.

### 2.6 Performance evaluation

Ensuring the consistency of model predictions with the outputs of the TPS is crucial. The mean absolute error (MAE) was used to evaluate the accuracy of dose predictions by the DL model (Equation 4). Additionally, significant attention was given to the relative volume of the rectum wall receiving 6300 cGy.

(4) $$\mathrm{MAE}\;=\frac{1}{N}\frac{\sum_{j=1}^{N}\frac{1}{M}\sum_{i=1}^{M}\left|D_{\mathrm{Pred}}\left(i\right)-D_{\mathrm{GT}}\left(i\right)\right|}{\mathrm{Prescription}\;\mathrm{dose}}*100\%$$

where *i* is the index of the voxel in each ROI for each patient, and *M* is the total number of the involved voxels. *D*_Pred_(i) and *D*_GT_(i) are the predicted and GT dose of a voxel *i*, respectively, *j* is the index of the case, and *N* is the total number of cases in the test set.

Subsequently, the prediction results of the DL model were compared with the outputs from the TPS. The model's prediction accuracy was assessed by calculating the differences between these two sets of results.

NTCP values generated from both the DL model and TPS were calculated separately. The difference between these two sets was used to evaluate the DL model's ability to assess clinical complications. The DL model's prediction results were input into the NTCP model to calculate corresponding risk values for complications. These results were then compared with those obtained by inputting TPS data into the NTCP model. By analyzing the discrepancies between these two results, the accuracy of the DL model in predicting complication risks could be evaluated.

To assess the accuracy of the decision‐making results, the outcomes from the dose prediction model were analyzed, using the TPS selection results as the ground truth. Receiver operating characteristic (ROC) curve and area under the curve (AUC) analyses were performed on these outcomes, using formulas (5), (6), and (7).

(5) $$\mathrm{SPE}=\frac{\mathrm{TN}}{\mathrm{FP}+\mathrm{TN}}$$

(6) $$\mathrm{SEN}=\frac{\mathrm{TP}}{\mathrm{TP}+\mathrm{FN}}$$

(7) $$\mathrm{AUC}=\int \mathrm{SEN}\left(1-\mathrm{SEN}\right)d\left(1-\mathrm{SEN}\right)$$

where the sensitivity (SEN) represents the proportion of all positive samples that are correctly predicted as positive samples, and the specificity (SPE) represents the proportion of all negative samples that are correctly predicted as negative samples. A positive result means the selection of proton therapy, while a negative result means the adoption of photon therapy.

### 2.7 Statistical analysis

*V*_6300cGy_ of the rectum wall and NTCP were subjected to statistical analysis. The normality of the samples and equality of variances were tested. The parametric paired *t*‐test with a two‐tailed approach was adopted to evaluate whether the difference between TPS and DL was statistically significant at *p* < 0.05(Table 3).

**TABLE 3:** MAE (mean ± standard deviation) of photon/proton dose prediction for target and OARs by the DL model. Lower MAE values mean that the predicted results are closer to the GT, indicating a better prediction performance of the model.

	Photon	Proton
ROIs	MAE (%)	MAE (%)
PTV1	1.88 ± 1.31	2.05 ± 0.87
PTV2	1.89 ± 1.40	2.10 ± 0.85
Rectum wall	4.60 ± 1.80	3.64 ± 1.27
Rectum	5.03 ± 2.10	3.74 ± 1.43
Bladder	2.98 ± 1.15	2.59 ± 1.17
Femur L	2.70 ± 1.11	3.36 ± 1.50
Femur R	2.82 ± 1.20	3.42 ± 2.15
Colon	2.85 ± 4.22	2.12 ± 3.70
Intestine	2.11 ± 1.68	0.80 ± 0.82

## 3 Results

### 3.1 Dose prediction accuracy

**FIGURE 3:**
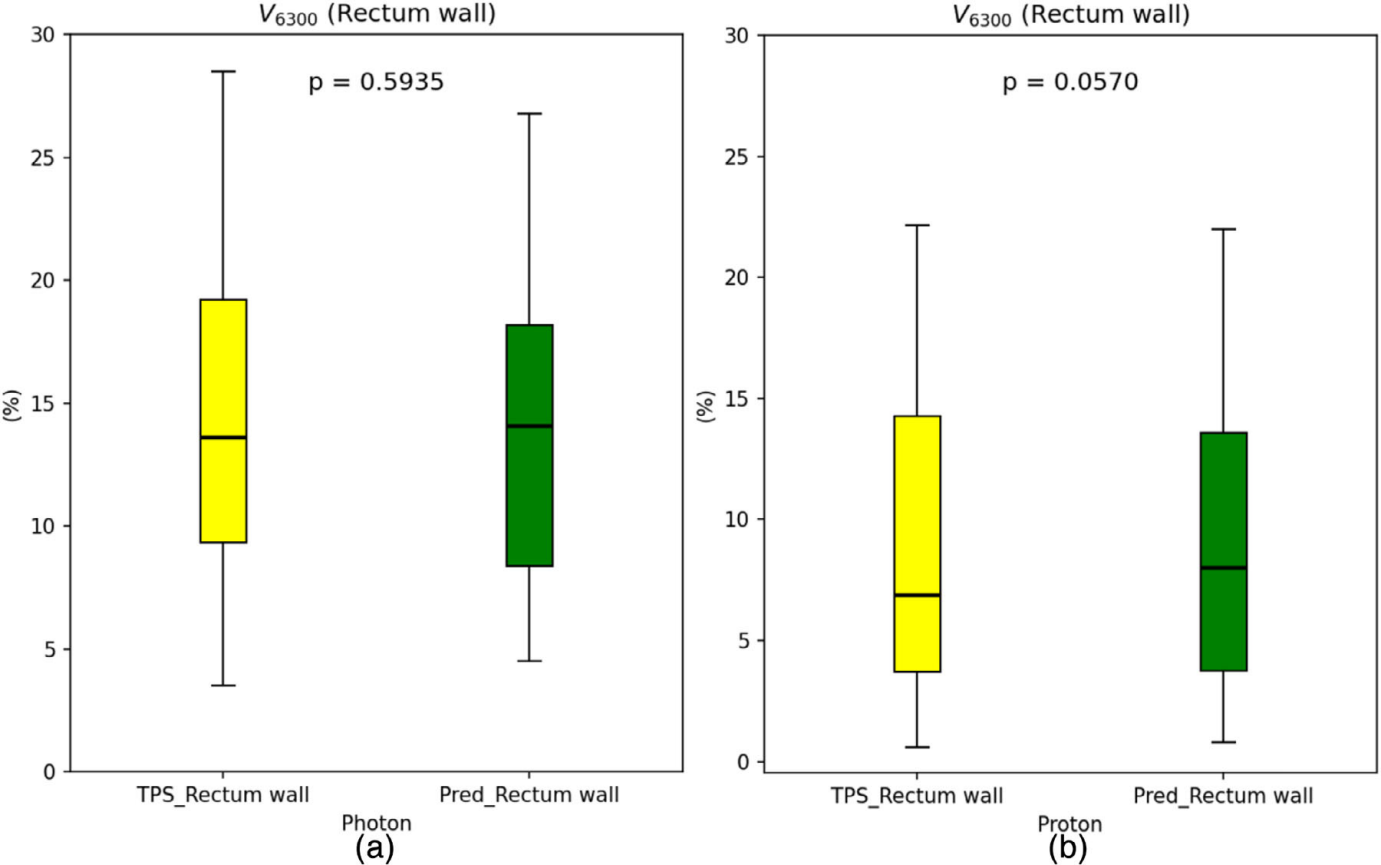
Box‐and‐whisker plots comparing the relative volume of rectum wall receiving 6300 cGy from TPS (left) and DL‐model prediction (right) are shown. Subfigure (a) represents the photon group, while (b) denotes the proton group. The boxes represent the interquartile range, with the median value indicated by a central line. Outliers are shown as individual points, and *p*‐values from parametric *t*‐tests are overlaid.

The MAE of various regions of interest (ROIs) in both photon and proton plans exhibited excellent performance, residing at a relatively low level compared to similar models^25^. For *V*_6300cGy_ (rectum wall), no statistically significant difference was observed between the prediction results of the DL model and TPS results (*p* = 0.5935 for the photon group and *p* = 0.0570 for the proton group; Figure 3).

### 3.2 NTCP model accuracy

**FIGURE 4:**
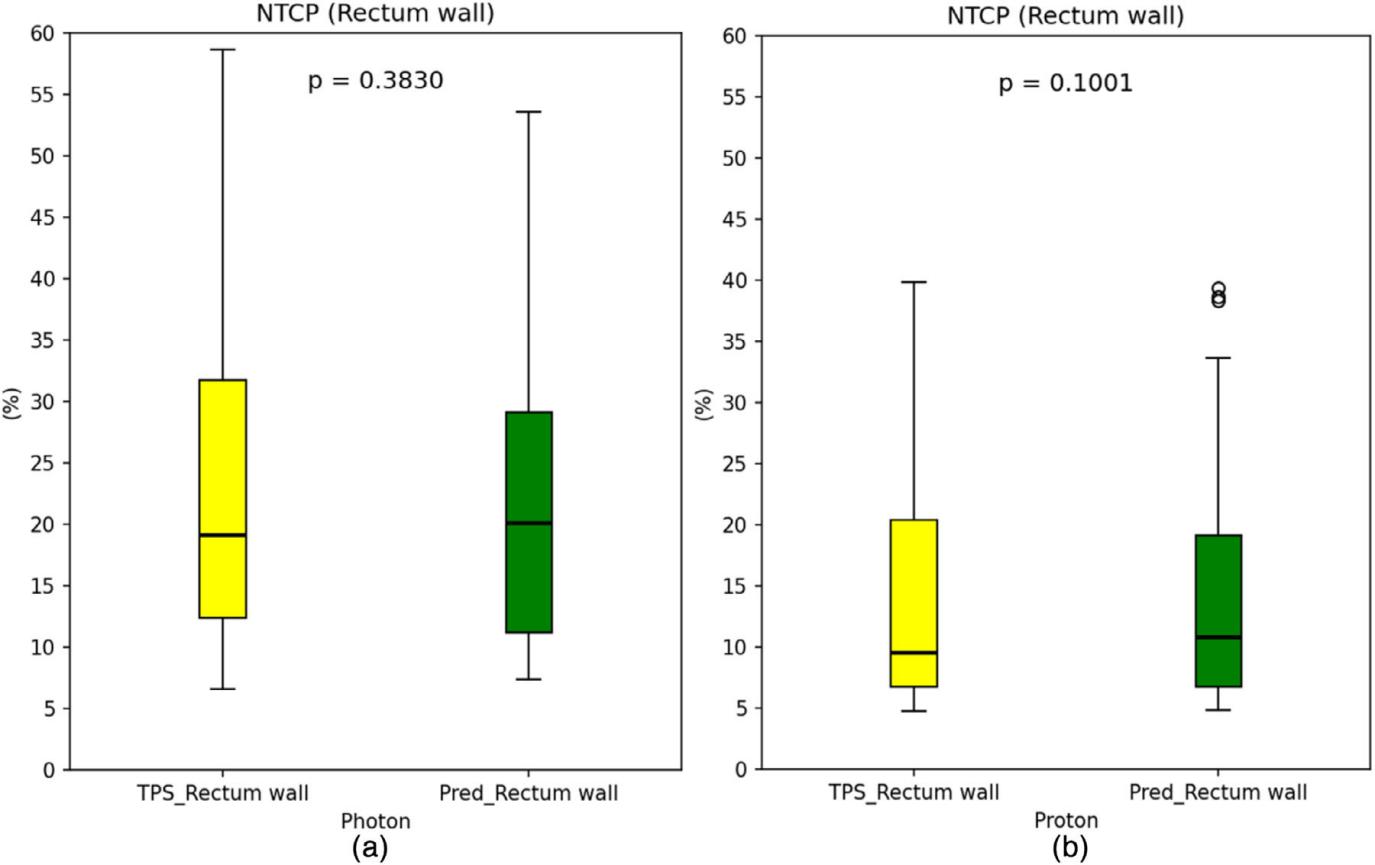
The NTCP of the rectum wall, derived from both TPS and DL model predictions, is represented using box‐and‐whisker plots. Subfigure (a) corresponds to the photon group, while (b) represents the proton group. The boxes indicate the interquartile range (25^th^–75th percentiles) and are intersected by the median value. Outliers are depicted as individual data points, with overlaid *p*‐values obtained from parametric *t*‐tests.

Figure 4 presents a comparison of NTCP between TPS and the model for the photon group (a) and the proton group (b). For photon‐based NTCP, the difference between TPS and the prediction was minimal, with a deviation of only 0.79 and a *p*‐value of 0.383. Similarly, for proton‐based NTCP, the values obtained from TPS (13.72 ± 9.45) and the prediction (14.62 ± 9.80) show strong concordance, as indicated by a *p*‐value of 0.100, reflecting favorable agreement.

### 3.3 Decision‐making accuracy

**FIGURE 5:**
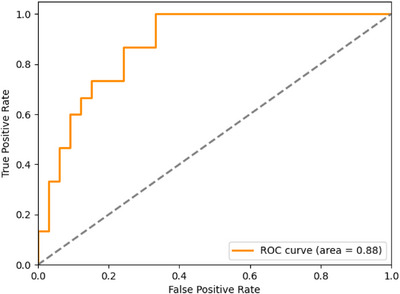
The analysis of the ROC curve for decision result prediction yielded an (AUC) value of 0.88. The closer the AUC to 1, the higher the accuracy of the decision‐making.

Among 48 patients, RT plans for 45 patients were correctly determined, resulting in an accuracy rate of 93.75%. The AUC value for the decision method was 0.88, comparable to the AUC reported in previous studies (Figure 5).^26^, ^27^, ^28^ The DL network incorrectly assigned treatment modalities for 3 patients. These three errors exhibited difference during the two‐step threshold approach.

In the first decision step, the photon NTCP values calculated by TPS for the two error cases were 13.61% and 12.71%, while the corresponding DL‐based prediction NTCP values were 9.95% and 9.10%, respectively, both close to the 10%. For the third case, the TPS‐calculated photon NTCP was 8.40%, whereas the DL‐predicted value was significantly higher at 21.21%.

In the second decision step, the ΔNTCP values computed based on TPS for the two cases are 7.70% and 7.61%, while the corresponding DL‐based ΔNTCP values are 10.50% and 11.84%, also near the 10%. For the third error case, the TPS‐based ΔNTCP value is 6.60%, while the DL‐predicted ΔNTCP is high at 19.45%.

## 4 Discussion

In this study, a database of photon and proton RT plans was established within our institution, and the feasibility of using the ResNet101 model for dose prediction in both modalities was validated. Additionally, a practical automatic decision‐assistance tool for prostate RT planning was developed.

The decision‐assistance tool provides patients with multiparameter comparison tables displaying NTCP values, dosimetric parameters, and treatment costs.

This automatic decision‐assistance tool allows for the pre‐evaluation of the most suitable RT technique for prostate cancer patients before treatment planning begins. It reduces the risk of complications related to inappropriate techniques. The method requires only the input of patient imaging information and RT structures into the model, with results generated automatically. It imposes no additional workload on clinical staff and does not necessitate changes to existing workflows. It provides both patients and doctors with an intuitive understanding of NTCP and recommendations for treatment selection.

The proposed automatic decision‐assistance tool has significant clinical implications. By employing appropriate treatment technologies that minimize normal tissue complications, it contributes to higher quality of life.^29^, ^30^

The accuracy rate of this decision‐assistance tool is 93.75%, with an AUC value of 0.88. Photon plans were selected for both TPS and DL in 31 cases. These patients typically have uniform body shapes, without implants of extreme densities. The PTVs in these cases are relatively regular, and the lymphatic drainage regions are small, which facilitates more precise predictions and outcomes. Proton plans were selected for both TPS and DL in 14 cases. The PTVs in these cases have pronounced concave and convex edges, which are closer to OARs. The Bragg peak characteristic of PT enables it to achieve steeper dose gradients than photon therapy.

In cases of incorrect decision‐making, where ΔNTCP values approach the 10%, decisions can vary even with minimal discrepancies between dose predictions and TPS results. The small volume of dose deposition in the rectum wall and the steep dose gradients can lead to inaccurate predictions of *V*_6300cGy_. The choice of ΔNTCP threshold significantly impacts the final decision, consistent with previous studies.^31^, ^32^ Additionally, the NTCP model used in this study was not specifically designed for PT cohorts, which may contribute to the incorrect decisions.

Currently, there is limited global research on decision‐making processes between proton and photon radiation therapy for prostate cancer. This article introduces a fully automated method that effectively aids in decision‐making for prostate cancer patients. The NTCP predictions obtained through this method are accurate and efficient compared to manual methods, validating its practical application. To enhance model applicability and flexibility, multiple dose gradient prescriptions have been incorporated, and addressing various uncertainties in the treatment process.

While PT offers significant advantages for prostate cancer treatment, photon therapy remains a mainstream option. Therefore, further investigation into the model's applicability to proton data is needed to provide decision support for a broader patient population. Additionally, the DL dose prediction model could be transferred from expert centers to less experienced ones, facilitating knowledge dissemination and improving access to advanced treatment planning capabilities. This characteristic is crucial for enhancing the overall level and coverage of medical services.

The limitation of this study lies in adopting NTCP—originally optimized for proton therapy—as the core metric for the decision‐assistance tool, with the accuracy of the NTCP model still needing improvement. Additionally, the substantial economic costs of PT and its clinical benefits, along with their impact on patients and their families, have not been evaluated. Future work will establish larger patient cohorts to develop photon‐specific NTCP models, enhancing the robustness of this study by simultaneously refining NTCP model accuracy and incorporating health economic assessments, while further validating our framework through multicenter studies.

Despite this, the NTCP model shows strong adaptability and versatility. By using alternative training data and transfer learning techniques, the model can be adapted to other tasks, opening up opportunities for further development and application. Future research should explore an end‐to‐end DL NTCP model that integrates 3D dose maps, organ anatomy, and clinical data to enhance prediction accuracy by directly using 3D information.^33^ This innovative approach is expected to provide more precise and personalized treatment plans for prostate cancer and other diseases.

## 5 Conclusions

We developed a fully automated decision‐assistance tool for selecting patients for proton therapy. This involves training a DL dose prediction model and utilizing an NTCP model to compute the probability of rectal complications. The system can generate a multiparameter comparison for patients, presenting NTCP decision outcomes, dosimetric metrics, and treatment costs, helping them choose the most suitable treatment approach. This tool holds significant and potential clinical practice value. Further multicenter validation with large‐scale cohorts is required to ensure model accuracy for clinical application.

## AUTHOR CONTRIBUTIONS

All authors have made substantial contributions to the work and development of this manuscript. All authors approved the manuscript. Mengyang Li and Linyi Shen designed and performed experiments, and completed the manuscript. Xinyuan Chen, Guiyuan Li, and Jialin Ding participated in measurements, scientific discussions, and manuscript writing. Kuo Men participated in scientific discussions and helped with manuscript writing. Junlin Yi and Jianrong Dai supervised the study and helped with writing of the manuscript.

## CONFLICT OF INTEREST STATEMENT

The authors declare no conflict of interest.
